# Depression in children with asthma: Testing a cholinergically mediated “endotype”

**DOI:** 10.1016/j.jacig.2025.100581

**Published:** 2025-10-10

**Authors:** Heather K. Lehman, Beatrice L. Wood, Quratulain Humayun, Weyman Lam, Bruce D. Miller

**Affiliations:** a Department of Pediatrics, Jacobs School of Medicine and Biomedical Sciences, State University of New York at Buffalo, Buffalo, NY; b Department of Psychiatry, Jacobs School of Medicine and Biomedical Sciences, State University of New York at Buffalo, Buffalo, NY; c Child and Family Asthma Studies Center, Buffalo, NY; d Warren Clinic, Saint Francis Health System, Tulsa, Okla

**Keywords:** Pediatric asthma, depression, cholinergic, vagal bias, anticholinergic medications, asthma endotype

## Abstract

**Background:**

Asthma is not a single uniform disease; instead, it comprises various disease sub entities, or “endotypes,” with different etiologies and pathophysiologies. As asthma endotypes are discerned, individuals with asthma can be matched with optimal therapies for their specific asthma subtype. Depression is common in persons with asthma and associated with increased asthma-related morbidity and mortality. Depression in child asthma is associated with a predominance of parasympathetic/cholinergic activation over sympathetic activation. Cholinergic activity mediates airway smooth muscle constriction and has effects on airway inflammatory cells and mucus-secreting goblet cells.

**Objective:**

The goal of the current study was to examine whether children with asthma who have more depressive symptoms have cholinergically mediated disease that is differentially responsive to short-acting anticholinergic therapy.

**Methods:**

A total of 39 children with asthma (aged 7-17 years) and baseline obstruction on spirometry were evaluated for depressive symptoms by using the Children’s Depression Inventory. Spirometry was used to evaluate the children for improvement in airway function following treatment with a short-acting anticholinergic medication. Inflammation was assessed by determining fractional exhaled nitric oxide levels and peripheral blood eosinophil counts, and atopic status was assessed by skin prick testing and measurement of total serum IgE level.

**Results:**

There was a statistically significant positive correlation between depressive symptoms and increase in FEV1 percent predicted value in response to ipratropium administration (*rs* = 0.344; *P* = .032).

**Conclusion:**

Higher depressive symptom scores are associated with increased airway responsiveness to inhaled ipratropium in children with asthma and active airway obstruction. These findings suggest the potential for definition of an endotype of asthma with comorbid depression that may benefit from anticholinergic asthma therapies.

Asthma affects more than 26 million people in the United States, including approximately 4.5 million children.^1^ Asthma is the most prevalent chronic disease of childhood, diagnosed in 6.5% of children.^2^ Despite advances in asthma therapies in the past several decades, asthma still carries significant morbidity and even mortality. In 2019, there were more than 1.8 million emergency room visits for asthma and about 170,000 inpatient admissions. In 2021, approximately 3,500 people died of asthma or as the result of complications associated with asthma.^1^

One possible obstacle to achieving better asthma outcomes may be that treatments are not sufficiently specific to asthma type. It is well accepted that asthma is not a single uniform disease but instead comprises many different disease subentities with different etiologies, pathophysiologies, and clinical manifestations. The term *endotype* was coined to delineate subtypes of asthma with distinct functional or pathophysiologic mechanisms.^3^ The division of asthma into endotypes is critical to the development of more specifically targeted treatment modalities to more effectively treat the heterogeneous group of patients encountered in clinical practice.

Adults and children with asthma have higher levels of depressive symptoms than do persons with most other chronic medical illnesses. Rates of current depressive disorders in outpatients with asthma have been estimated to range from 14% to 25%, and 31% to 47% of patients with asthma have had depression at some point in their lives.^4^, ^5^, ^6^ In a meta-analysis conducted by Gao et al, depression was associated with a 43% increased risk of developing adult-onset asthma.^7^ Depression is associated with unfavorable asthma outcomes, including medication nonadherence, increased frequency of emergency room visits, hospitalizations, unscheduled visits for asthma,^8^ and even asthma-related death.^9^^,^^10^ Morrison et al found that about 30% of children with asthma had scores on the Children’s Depression Rating Scale-Revised consistent with major depressive disorder, and depressive symptom severity in these children was associated with a greater number of hospitalizations.^11^ Waxmonsky et al showed that depression severity was associated with level of asthma disease activity in children.^12^ Although worse outcomes may be related to behavioral factors such as medication nonadherence, there is evidence to suggest that there may be a direct depression-related psychobiologic mechanism contributing to this phenomenon as well.

There is a substantial body of evidence in humans and experimental animals linking depression and depressive emotional states to increased parasympathetic activity and cholinergic/vagal tone.^13^, ^14^, ^15^, ^16^, ^17^, ^18^ On the basis of this research and clinical observation, Miller proposed the autonomic dysregulation theory of depression and asthma.”^19^ This theory proposes a pathophysiologic mechanism of depression-related asthma. Specifically, it posits that depression is associated with excess parasympathetic/vagal activation as compared with sympathetic activation in response to stress, which in turn activates cholinergic muscarinic receptors on bronchial smooth muscle, thus leading to bronchoconstriction.^20^ Using an experimental laboratory paradigm to study children with asthma, Miller^19^ demonstrated that those children with greater depressive symptom severity had increased parasympathetic/vagal activation relative to sympathetic activation (ie, vagal bias) while observing emotionally challenging movie scenes. This parasympathetic activation was associated with greater airway resistance. In contrast, children with asthma and a low level of depressive symptoms showed increased sympathetic activation relative to parasympathetic activation when experiencing these same emotionally challenging scenes.^21^

An inhaled corticosteroid (ICS) medication, either alone or in combination with a long-acting β-adrenergic (LABA) is the recommended controller therapy for patients with persistent asthma. Although effective for many patients, ICS therapy may be less effective for those whose asthma is not induced primarily by eosinophilic inflammation.^22^^,^^23^ Anticholinergic medications may be effective as adjunctive therapy in patients with asthma that is not well controlled by an ICS with a LABA.^24^ One study has demonstrated a larger bronchodilatory response to inhaled anticholinergics in patients with nonallergic asthma than in those with allergy-mediated asthma, whereas another study has supported the efficacy of anticholinergic therapy in patients with asthma without eosinophilic inflammation.^25^^,^^26^

These previous studies demonstrating the efficacy of anticholinergics in nonallergic, noneosinophilic asthma did not assess for comorbid depression as a potential factor influencing cholinergically mediated airway constriction. In adults with asthma, major depression has been associated with decreased bronchodilatory response to albuterol, which is a β-adrenergic bronchodilator.^27^

The purpose of the present study was to examine the plausibility of a novel, depression- related, cholinergic-mediated “endotype” of asthma that may benefit from inhaled anticholinergic medicines in a rescue and controller asthma regimen. Several findings would be anticipated if depression-related asthma were a distinct asthma endotype: (1) the level of depressive symptomatology in children with asthma would be associated with the degree of positive airway response to ipratropium, as measured by spirometry; (2) there would be an inverse association between level of depressive symptomatology and residual response to albuterol administered after inhaled ipratropium; and (3) the level of depressive symptomatology would be inversely associated with the level of atopy or inflammation.

## Methods

### Recruitment

This study was approved by the University at Buffalo’s institutional review board. Study subjects were recruited from a population of patients of UBMD Pediatrics subspecialty clinics. Potential subjects were either approached in person by the study team during a routine clinic visit at UBMD Pediatrics or sent an opt-out letter via mail, followed by a phone call 2 weeks after the letter had been mailed. To qualify on the initial chart screen, patients must have been diagnosed with asthma by a physician and must have been between 7 and 17 years of age at the time of participation in the study.

### Procedure

On the day of the study visit, informed consent was obtained and subjects were screened for eligibility based on inclusion and exclusion criteria. The inclusion criteria were as follows: (1) age 7 to 17 years; (2) physician-diagnosed asthma; and (3) decreased lung volumes for age, height, and/or race (FEV_1_ value ≤ 80% predicted; ratio of FEV_1_ value to forced vital capacity (FVC) no higher than 85%; or at least a 12% decrease in FEV_1_ compared with personal best in chart) on the day of study visit, as assessed by portable spirometry.

The exclusion criteria were as follows: (1) inability to successfully perform the spirometry maneuver, (2) normal lung volumes at the time of the study visit (FEV_1_ value > 80% and FEV_1_/FVC ratio > 85%), (3) asthma exacerbation requiring systemic steroids in the week preceding the study visit, (4) pregnancy or nursing, (5) history of significant cardiopulmonary disease other than asthma, (6) history of conditions exacerbated by anticholinergic therapy, (7) current use of another anticholinergic medication, or (8) a non–English-speaking patient or caregiver who could not complete the study instruments.

Baseline spirometry was performed using a portable spirometer (Spirobank II Basic, MIR, Rome, Italy). Participants meeting the inclusion criteria continued in the study. Baseline spirometry (FEV_1_ percent predicted value) was recorded as the pre–ipratropium administration measure of airway function. Participants next answered a self-report questionnaire (the Children’s Depression Inventory [CDI]) assessing depressive symptoms. Then, they received 2 puffs of ipratropium with hydrofluoroalkane as a propellant (total of 42 μg of ipratropium) administered through a disposable spacer. During the next 30 minutes, skin prick testing to a panel of 17 perennial and seasonal allergens was used to assess atopic sensitization.

**Fig 1:**
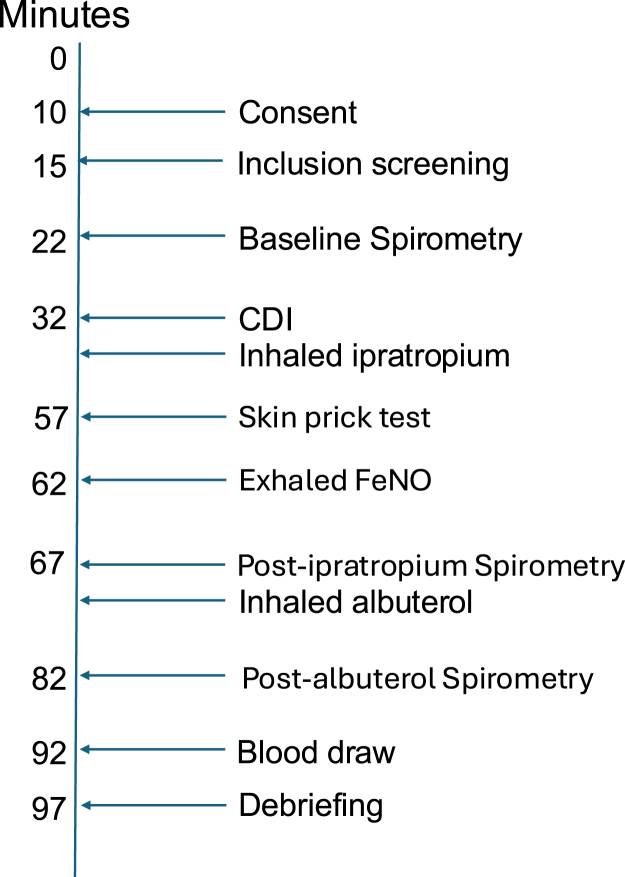
Study procedural timeline (times indicate completion time for individual steps of the study procedure, in minutes)

Fractional exhaled nitric oxide (Feno) level was then measured to assess eosinophilic lung inflammation by using a Feno measurement device (NIOX VERO, NIOX Group, Oxford, United Kingdom). Feno level was measured at least 30 minutes after the initial spirometry to prevent decreases in Feno level by the spirometry forced expiratory maneuver, which has been reported to occur in the 6 minutes following spirometry in children with asthma.^28^ Next, each subject performed spirometry after ipratropium administration, and FEV_1_ percent predicted was recorded. At this point each subject received 2 puffs of albuterol with hydrofluoroalkane as a propellant. After 15 minutes, spirometry was again performed to assess residual bronchodilation after albuterol. Finally, participants had blood drawn for measurement of their total serum IgE level and peripheral blood eosinophil count. Determination of serum specific IgE levels by ImmunoCAP was added to the laboratory workup if the patient was unable to undergo skin testing because of antihistamine use. All participants were assessed clinically by the study physician to ensure clinical stability and safety before leaving the study (Fig 1).

### Measures

Spirometry was performed using NHANES III (National Health and Nutrition Examination Survey III) reference equations to determine FEV_1_ percent predicted and FEV_1_/FVC ratio.^29^ This measure was used to determine eligibility for the study. Feno level was measured and reported in parts per billion (ppb).

The CDI was used to assess depressive symptoms. The CDI is a 27-item self-report scale for depression in children, with validated reliability and validity in children aged 7 to 17 years. Each item in the CDI is rated from 0 (no depressive symptoms) to 2 (clear presence of depressive symptoms).^30^^,^^31^ The version of the CDI used in this study removed the single question on self-harm at the request of the local institutional review board. Adjusted T scoring on the CDI allows interpretation across ages and sex.

Skin prick testing to 17 common allergens was used to assess atopic sensitization. Testing was specific to seasonal (tree, grass, and weed pollens) and perennial (mites, roaches, animal dander, and mold) allergens.^32^ A skin wheal diameter 3 mm greater than the saline control was interpreted as positive allergen sensitization. For participants undergoing antihistamine therapy at the time of study visit, levels of serum specific IgE^33^ to the same panel of seasonal and perennial allergens were obtained in place of skin prick testing.

### Data analysis

The data were examined for distribution and outliers by using the Shapiro-Wilk test of normality using coefficients of skewness and kurtosis. Descriptive statistics were calculated and reported as means and SDs for quantitative variables, such as age, CDI score, and change in FEV_1_ percent predicted, and also as frequencies and percentages for qualitative variables (sex, ethnicity, and race). The data were assessed with regard to statistical assumptions required for Pearson product-moment correlation; it was found that CDI did not meet the assumptions of normality. Therefore, as customary, the Spearman rho correlation coefficients were used to evaluate linear associations between variables.

## Results

A total of 80 subjects presented for a screening visit in office, signed consent and assents, and underwent baseline spirometry. In all, 39 of these subjects had evidence of obstruction on baseline spirometry, fulfilled all other inclusion and exclusion criteria, and were enrolled in the study. Baseline demographic characteristics of the 39 subjects are included in Table I. Baseline controller asthma medication therapy was assessed; 8 subjects did not use a daily asthma controller, 4 subjects used ICS controller therapy, 1 subject used only a leukotriene receptor antagonist for controller therapy, 3 subjects used an ICS and leukotriene receptor antagonist for controller therapy, 10 subjects used ICS/LABA controller therapy, and 10 subjects used ICS/LABA in combination with an asthma biologic. Two subjects’ baseline controller asthma therapy was not documented.

**Table I:** Demographic characteristics of the 39 study participants

Characteristic	No.	%
Sex		
Female	19	48.7
Male	20	51.3
Race		
American Indian/Alaskan Native	0	0
Asian	0	0
African American/Black	18	46.2
White	18	46.2
>1 race	2	5.0
Not reported	1	2.6
Ethnicity		
Hispanic/Latino	10	25.6
Non-Hispanic/Latino	29	74.4

The participant CDI T scores ranged from 37 to 78, with a mean of 47.46 (Table II). Of the 39 participants, 6 (15.4%) had scores of 55 or higher, indicating clinically significant depressive symptoms.

**Table II:** Baseline clinical assessment scored

Characteristic	Mean	SD
Age (y)	12.92	3.27
CDI (T score)	47.46	9.26
FEV_1_ percent predicted (baseline)	80.05	20.89
Feno level (ppb)	44.62	48.80
Blood eosinophil count (cells/mm^3^)	387.38	298.15
Specific allergen sensitization (no. of the 17 allergens for which a positive result was found)	3.06	3.08
Serum IgE level (IU/mL)	613.6	696.6

**Fig 2:**
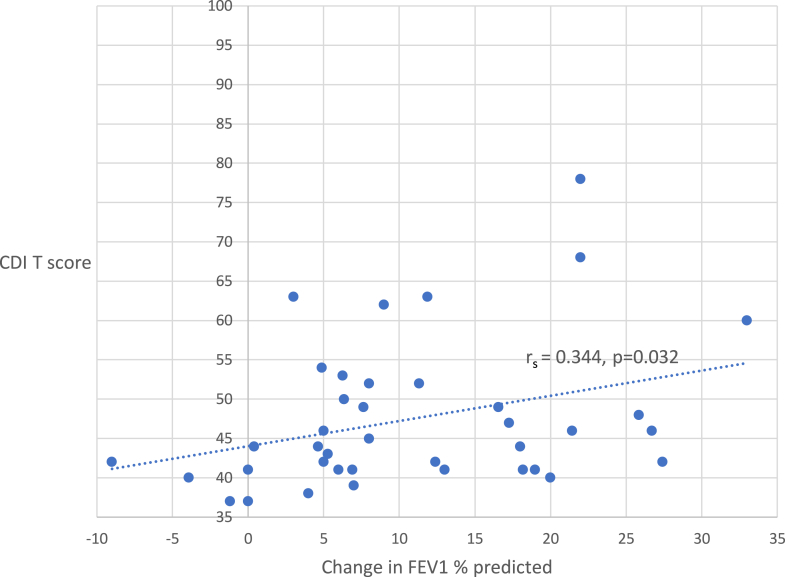
Correlation of depressive symptoms (CDI T score) with change in FEV_1_ percent predicted following ipratropium inhalation.

In the entire study sample, the mean change in FEV_1_ percent predicted following ipratropium administration was 10.8% (range –9% to +33% change). There was a statistically significant positive correlation between CDI T score and increase in FEV_1_ percent predicted in response to ipratropium administration (*rs* = 0.344; *P* = .032 [Fig 2]).

A total of 32 subjects received albuterol after post–ipratropium administration spirometry, and the average *additional* change in FEV_1_ percent predicted following albuterol administration was 8.9% (range –7% to +45%). There was no significant correlation between CDI T score and additional change in FEV1 percent predicted in response to albuterol administration (*rs* = –0.176; *P* = .334).

The means and SDs for peripheral blood eosinophil counts, total serum IgE level, Feno level, and specific allergen sensitization (by either skin prick test result or serum specific IgE level) are reported in Table II. There was no statistically significant relationship observed between the CDI T score and each of these immune and allergy parameters (Table III).

**Table III:** Correlation between CDI T score and immune/allergic marker

Marker	No.	Correlation with CDI T score (*r*_*s*_)	Significance (*P* value)
Blood eosinophil count (cells/mm^3^)	31	–0.289	.11
Feno level (ppb)	29	–0.05	.8
Specific allergen sensitization (no. of the 17 allergens for which a positive result was found)	35	0.086	.62
Serum IgE level (IU/mL)	29	–0.19	.32

## Discussion

No previous studies have systematically assessed whether patients with asthma plus depressive symptoms may be differentially responsive to anticholinergic asthma medications. In this study, we demonstrated, as hypothesized, that greater depressive symptomatology correlated with degree of positive airway response to ipratropium. There was no association between degree of depressive symptomatology and residual response to albuterol administered after ipratropium treatment. The level of airway response to ipratropium was not associated with degree of atopy (positive skin test result or serum IgE level) or inflammation (Feno level or peripheral blood eosinophil count).

These findings are consistent with our assertion that greater depressive symptoms in children with asthma are associated with increased cholinergically mediated bronchoconstriction and not simply with worsened asthma or more severely compromised lung function.

We did not find the predicted inverse relationships between depressive symptoms and the several assessed inflammatory or allergic markers, although a trend toward an inverse relationship with blood eosinophil count was seen. Our sample size was small, which may have limited the potential for finding significant relationships with depressive symptoms. Also, we did not have patients hold their controller medications before the study visit. Use of baseline controller medications may have blunted differences in inflammatory measures, especially Feno level, thus preventing observation of an association with depressive symptoms. Alternatively, because parasympathetic neuronal innervation affects signaling that modulates inflammatory cells and nonneuronal cells such as fibroblasts,^34^ the negative impact of depression on asthma airway function occurring through the parasympathetic/vagal autonomic nervous system pathway may actually amplify the baseline allergic asthma pathophysiology common in pediatric asthma, rather than acting as a separate alternative mechanism of airway compromise in asthma exacerbations.

Also, we did not find a significant inverse relationship between depressive symptoms and residual bronchodilatory response to albuterol, although there was a weak trend in this direction. By assessing residual bronchodilatory response to albuterol immediately after assessing bronchodilatory response to ipratropium rather than at a separate encounter, we may have limited our ability to detect subtle differences in response to albuterol related to depressive symptoms.

Previous studies have investigated immune pathways by which stress may affect asthma.^35^ However, this research does not specifically address the role of depressive symptoms, despite the fact that depression is highly associated with asthma morbidity. Our findings are consistent with those of other studies suggesting that vagal/cholinergic pathways are implicated in airway response to stressful emotions on asthma. These studies demonstrate the association of emotional stress with increased cholinergic activation and vagal tone in persons both with and without asthma.^36^, ^37^, ^38^ A controlled laboratory-based study demonstrated that ipratropium bromide blocked the effect of airway constriction in response to negative emotional stimuli.^39^ Results from these studies demonstrate that stressful negative emotions affect airway function and that these effects are mediated at least in part by cholinergic pathways. However, depressive symptoms were not examined in these studies.

Liccardi et al^40^ reported that 63% of 860 patients with asthma had co-occurring nonrespiratory symptoms (eg, anxiety, reflux, heartburn, stomach pain) preceding or accompanying their asthma attacks. They postulated that these asthma attacks may have resulted from imbalance between sympathetic/parasympathetic systems, specifically with an increase in parasympathetic cholinergic tone associated with these nonrespiratory conditions. They further reasoned that in some subsets of patients, including those with underlying depression, increased cholinergic tone might play a prevalent role potentiating the triggering of bronchospasm. They proposed this category of patients as belonging to a novel asthma phenotype—one indicating preferential efficacy of anticholinergic asthma intervention.^41^

Our current study derives from Miller’s Autonomic Nervous System Dysregulation Theory of Depression and Asthma.^19^^,^^20^ In accordance with this theory, Miller’s research demonstrates that in pediatric patients with comorbid depression and asthma, increased parasympathetic/cholinergic activation relative to sympathetic activation (ie, vagal bias) was associated with increased airway resistance in response to emotionally challenging movie stimuli. From their findings, they concluded that this vagal bias may be a specific mechanism affecting airway function in patients with comorbid asthma and depression.^20^^,^^21^^,^^42^, ^43^, ^44^

Findings from the present study extend the empirical research informed by Miller’s theory and support the supposition that comorbid depression and asthma may be a newly identified, potentially valid endotype in asthma—one that could potentially benefit from adjunctive anticholinergic treatment.

### Strengths, limitations, and future directions

This is the first study using a clinical experimental design to examine the plausibility of a novel, depression-related, vagal/cholinergically mediated endotype of asthma. The study extends previous research in support of a theory that depression affects airway function by way of the vagal/cholinergic pathway/mechanism. We posit that the findings herein are consistent with a depression-cholinergic endotype of asthma and that they lay the groundwork for more systematic and controlled examination of this entity.

There are limitations of the current study that call for further research. The sample size may have been insufficient to detect inverse associations of depressive symptoms with either allergic or inflammatory markers or with the bronchodilatory response to albuterol. For this preliminary study, we also did not have participants hold their asthma controller medications (including ICSs and ICSs-LABAs) before the study visit to minimize risk of loss of asthma control, as our study had no direct benefits to study participants. This approach may have decreased inflammatory markers, especially Feno level, in participants taking these baseline medications. Although use of a LABA as part of a controller regimen could blunt bronchodilatory response to ipratropium or albuterol, in our study there was no significant difference in change in FEV_1_ percent predicted following use of either bronchodilator when comparing subjects reporting use of a LABA-containing controller regimen with those subjects not taking a LABA. Future studies should assess the association of depressive symptoms with responses to ipratropium versus to albuterol administered in a within-subject crossover design conducted on separate experimental sessions rather than sequentially during the same study visit as done in our study. Future studies must include systematic control of potential confounders that were beyond the scope of the current study (ie, prescribed medications, including ICSs and LABAs), as well as medication adherence and potential albuterol overuse. Such precision requires larger, carefully controlled studies.

Extending this investigation to systematically examine race, ethnicity, and socioeconomic status factors will be important as well. Finally, the findings of our study suggest the need for larger studies to rigorously evaluate the validity of considering asthma and comorbid depression as a new endotype mediated by cholinergic activation.

### Conclusions

Despite recent advances in asthma therapeutics, many children and adults with asthma still have difficult-to-control disease. The failure to factor in the effect of depression on airway function may contribute to challenges in treating asthma effectively in these patients. Adjunctive anticholinergic medications are used in patients with severe asthma, but with limited understanding of which patients might benefit most from this add-on therapy. Our findings suggest that it may be beneficial to screen patients with asthma for depressive symptoms, as they have the potential to be a subset of patients who are particularly responsive to anticholinergic therapies. Larger-scale clinical trials are needed to test the potential therapeutic utility of using both short-acting and long-acting inhaled anticholinergic medications in patients with asthma and comorbid depression.

**Clinical implications**

Despite recent advances in asthma therapeutics, many patients still have difficult-to-control disease. Our findings suggest that children with asthma and depressive symptoms may be particularly responsive to anticholinergic asthma therapies.

## Disclosure statement

Supported by the 10.13039/100006108National Center for Advancing Translational Sciences of the 10.13039/100000002National Institutes of Health (award UL1TR001412 [to the 10.13039/100008209University at Buffalo]). The content is solely the responsibility of the authors and does not necessarily represent the official views of the National Institutes of Health.

Disclosure of potential conflict of interest: The authors declare that they have no relevant conflicts of interest.
